# Going Into a Community, We Need to Start From Where They’re At: Caregiver and Advocate Perspectives

**DOI:** 10.1093/geroni/igab046.1783

**Published:** 2021-12-17

**Authors:** Zachary Baker

**Affiliations:** University of Minnesota, University of Minnesota/Minneapolis, Minnesota, United States

## Abstract

We recorded and inductively coded an open-ended discussion of jargon surrounding “dementia” with the “Supporting Dementia Caregivers After Death” community advisory board (CAB). CAB-members included current and former caregivers of PLWD due to early- and normal-onset Alzheimer’s, Lewy body, and Parkinson’s, a co-president of the Alzheimer’s Association (ALZ) Young Champions, a dementia trainer/consultant and member of a Catholic church that preserves American Indian spiritual traditions, a senior program manager at ALZ who was entrusted by American Indian reservation elders to provide dementia education, a care partner support group leader, and an Alzheimer’s Ambassador chosen by multiple US senators. Themes identified included differential inclusiveness of terms like “memory loss” versus “dementia”, misuse and misunderstanding of “dementia” versus “Alzheimer’s,” and the difficulty of translating “dementia” into the American Indian Ojibwe (i.e., Anishinaabemowin) language where suggested translations directly translated to “slow memory loss”, “brain deterioration”, “absent mindedness”, or even “craziness”.

